# Predicting locoregional treatment failure in anal cancer using multivariable clinical-imaging models

**DOI:** 10.1016/j.ctro.2026.101187

**Published:** 2026-05-17

**Authors:** C. Ceuppens, L.A. Min, B. van Triest, M. Lopez-Yurda, W.V. Vogel, P. Snaebjornsson, M. Maas, R.G.H. Beets-Tan, G.L. Beets, B.A. Grotenhuis, D.M.J. Lambregts

**Affiliations:** a Department of Surgery, Netherlands Cancer Institute, Amsterdam, the Netherlands; b GROW School for Oncology and Reproduction, University of Maastricht, Maastricht, the Netherlands; c Department of Radiology and Nuclear Medicine, Gelre Ziekenhuizen, Apeldoorn, the Netherlands; d Department of Radiation Oncology, Netherlands Cancer Institute, Amsterdam, the Netherlands; e Department of Biometrics, Netherlands Cancer Institute, Amsterdam, the Netherlands; f Department of Nuclear Medicine, Netherlands Cancer Institute, Amsterdam, the Netherlands; g Department of Pathology, Netherlands Cancer Institute, Amsterdam, the Netherlands; h Faculty of Medicine, University of Iceland, Reykjavik, Iceland; i Department of Radiology, Netherlands Cancer Institute, Amsterdam, the Netherlands; j Maastricht Radiation Oncology (MAASTRO), Maastricht, the Netherlands; k Department of Surgery, Maastricht University Medical Centre, Maastricht, the Netherlands

**Keywords:** Anal squamous cell carcinoma (ASCC), Chemoradiotherapy, (18F-)FDG-PET/CT, Outcome prediction

## Abstract

**Background and purpose:**

Concurrent chemoradiotherapy (cCRT) is an effective treatment for anal squamous cell carcinoma, but identifying patients with good treatment outcomes remains challenging. We aimed to develop a multivariable model combining baseline clinical and imaging (MRI, PET/CT) variables, HPV status and semi-quantitative 18F-FDG-PET/CT parameters to predict locoregional treatment failure, defined as incomplete response or local relapse, within two years after cCRT.

**Materials and methods:**

This retrospective cohort study included 152 anal cancer patients treated with cCRT between 2008 and 2023. All patients underwent pre-treatment 18F-FDG-PET/CT and ≥ 2 years of follow-up was available. Seven predictive models were constructed using combinations of (1) clinical baseline variables (age, sex, cTN-stage), (2) tumour hr-HPV status, and (3) PET/CT parameters (e.g. SUVmax, MTV42%, TLG). Model performance for predicting locoregional failure was evaluated using bias-corrected area under the receiver operating characteristic curve (AUC), using LASSO regression.

**Results:**

Locoregional failure occurred in 21 of 152 patients (14%) within two years after cCRT. The model combining hr-HPV and PET/CT parameters showed the best performance (bias-corrected AUC 0.69). In this model, hr-HPV positivity was associated with substantially lower odds of failure (OR 0.30, 95% CI 0.07–1.00), while higher MTV42% was associated with slightly increased odds (OR 1.01, 95% CI 1.00–1.05).

**Conclusion:**

The model combining hr-HPV status and semi-quantitative PET/CT parameters (particularly MTV42%) performed moderately well to predict two-year locoregional failure in anal cancer patients. These findings support further exploration of baseline risk stratification to develop models that may guide more personalised treatment strategies.

## Highlights

• Predicting locoregional treatment outcomes in anal cancer remains challenging.
• Hr-HPV positive tumours have substantially lower odds of locoregional failure.
• A model combining hr-HPV status and PET-variables predicts failure moderately well.
• Our findings support further exploration to guide personalised treatment strategies.

## Introduction

Anal squamous cell carcinoma (ASCC) or simply ‘anal cancer’ is a rare malignancy, although its global incidence is rising, mainly due to the increased prevalence of high-risk human papillomavirus (hr-HPV) which is strongly associated with ASCC [1], [2], [3]. The standard treatment for most anal cancers – cT2-4 tumours and cT1 tumours measuring ≥ 1 cm – is concurrent chemoradiotherapy (cCRT) with curative intent, which results in a complete clinical response in the majority of patients [4], [5], [6], [7]. Despite this, locoregional treatment failure (i.e. residual or recurrent disease at the primary tumour site or regional lymph nodes) occurs in approximately 15–30% of patients, predominantly within two years after start of treatment [8], [9], [10]. In these cases, salvage surgery – typically an abdominoperineal resection (APR) with definitive colostomy – is the next treatment step, provided that a radical resection is deemed feasible. These procedures can be extensive, carry a substantial risk of complications, and have a large impact on patient’s quality-of-life [[11], [12], [13]].

Based on the recently published PLATO ACT-4 data, discussions within national guideline committees are increasingly supporting dose de-escalation in patients with early-stage anal cancer (T1-2 N0-1, tumour < 4 cm) [14]. In this context, it would be valuable to identify, prior to treatment, which patients are likely to respond well, in order to better estimate the chance of successful dose de-escalation. Conversely, early identification of patients at increased risk of locoregional failure could guide the selection of tailored treatment strategies, such as radiation dose escalation (boost), intensified chemotherapy or novel systemic therapies, aimed at improved cure rates while preserving organ function [15]. Several clinicopathological factors have already been associated with an increased risk for treatment failure, including larger tumour size, node-positive disease, male sex and hr-HPV negative status [[16], [17], [18]]. However, these variables lack sufficient discriminatory power when used individually, and validated multivariable models are not yet available. Therefore, the development of improved risk stratification strategies is increasingly recognised as a priority within international collaborative research initiatives [19].

Fluorodeoxyglucose positron emission tomography/computed tomography (18F-FDG PET/CT) is increasingly used in the pre-treatment work up of anal cancer for staging, treatment stratification and radiotherapy planning [20]. In addition to its diagnostic role, semi-quantitative PET/CT-derived parameters, such as standardised uptake value (SUV) and metabolic tumour volume (MTV), have shown potential as predictors of treatment response, disease recurrence and survival [[21], [22], [23], [24], [25], [26]]. However, many of these studies are limited by small or heterogeneous patient cohorts, and very few have integrated PET/CT parameters with clinicopathological factors to build comprehensive clinical prediction models.

The aim of this study was to develop a multivariable model combining baseline clinical and imaging (MRI, PET/CT) staging variables, hr-HPV status and semi-quantitative 18F-FDG-PET/CT parameters to predict locoregional treatment failure in a homogenous cohort of anal cancer patients treated with cCRT.

## Materials and methods

**Fig. 1:**
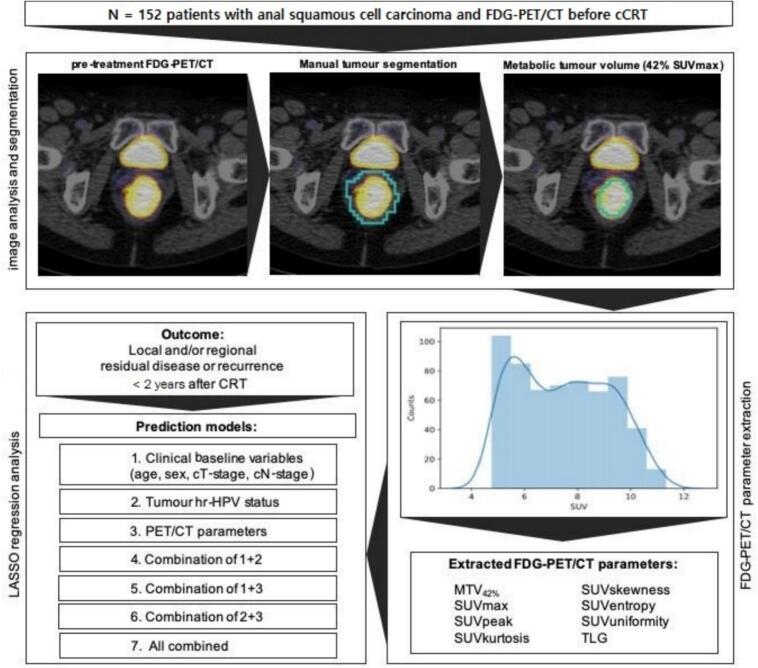
Schematic outline of the study and analysis workflow.

A schematic outline of the study workflow is provided in Fig. 1.

### Study design and patient selection

This retrospective cohort study was conducted at the Netherlands Cancer Institute and approved by the Institutional Review Board. Patients treated with curative intent for primary anal cancer between 2008 and 2023 were identified. Inclusion criteria were:

1. Histopathologically confirmed anal squamous cell carcinoma;
2. Availability of tumour hr-HPV status
3. No distant metastases;
4. Pre-treatment 18F-FDG-PET/CT with visible primary tumour in situ;
5. Treatment with standard cCRT protocol (see below);
6. Minimum of two years of post-treatment follow-up.

### Treatment protocol

Radiotherapy was administered using intensity-modulated radiotherapy (IMRT) or volumetric-modulated arc therapy (VMAT), with a simultaneous integrated boost (SIB) of 59.4 Gray (Gy) to the gross tumour volume (GTV) and 49.5 Gy to elective nodal regions. Chemotherapy consisted of capecitabine (825 mg/m^2^ twice daily on radiotherapy days) or 5-fluorouracil (750 mg/m^2^ daily for five consecutive days during weeks 1 and 5). Mitomycin C (12 mg/m^2^, maximum 20 mg) was administered on the first day of treatment. Patients with macroscopic residual disease at week 5 (assessed by digital rectal examination and/or MRI) received an additional sequential boost of 3 × 1.8 Gy on the primary tumour (total dose 64,8 Gy).

### Outcome measures

The primary outcome was 2-year locoregional failure, defined as residual or recurrent disease after completion of cCRT within the radiation field, including the primary tumour site and locoregional lymph node regions. Residual disease was defined as the presence of persistent tumour and/or suspicious lymph nodes following completion of concurrent chemoradiotherapy (cCRT), without evidence of a clinical complete response during the first 6 months after completion of cCRT. Recurrent disease was defined as the reappearance of tumour or lymph node metastases after an initial clinical complete response, as determined by post-treatment MRI, digital rectal examination and endoscopic evaluation. Surveillance included clinical examination every three months, serum SCC antigen testing, and imaging when indicated. Residual or recurrent disease was confirmed by histopathology of surgical specimens and/or biopsies, or − when tissue confirmation was unavailable − by concordant clinical, MRI and 18F-FDG-PET/CT findings.

### Clinical baseline variables

Clinical baseline variables included age, sex, tumour length and clinical T- and N-stage (cTN stage), classified according to the UICC TNM Classification of Malignant Tumours, 8th edition (2017). Maximum tumour length was retrieved from the MRI staging reports and cTN stage was based on multidisciplinary team assessments integrating physical examination, MRI, 18F-FDG-PET/CT and ultrasound ± fine-needle aspiration. Tumour hr-HPV status was retrieved from institutional pathology records and status was determined by polymerase chain reaction (PCR) and/or p16 immunohistochemistry with p16 being an established surrogate biomarker [27]. Although serum SCC antigen was measured as part of routine post-treatment surveillance, baseline pre-treatment values were not consistently available across the cohort and were therefore not included as a predictor variable in the analyses.

### PET/CT acquisition and extraction of semi-quantitative PET variables

PET/CT scans were performed using Gemini TF 16 or TF Big Bore scanners (both Philips Healthcare, Best, Netherlands). Patients fasted for ≥ 6 h prior to intravenous administration of 180 or 280 MBq of 18F-FDG (depending on body mass index). Scanning ensued after 60 +/- 10 min of rest, from skull base to mid-thighs, with 2 min per bed position. PET images were attenuation corrected using native CT images (acquired at 120–140 kV, with 29–324 mAs using automatic dose-modulation) and reconstructed to 4 × 4 mm pixels and 4 mm slices. CT images were reconstructed to 1.17 × 1.17 mm pixels and 2 mm slices with a 2 mm slice spacing. Images were transferred to an offline workstation for semi-automatic tumour segmentation using the open-source software 3D Slicer (v4.8.1, slicer.org). PET/CT parameters were subsequently extracted using PyRadiomics (version 2.2.0, pyradiomics.readthedocs.io) [28]. A preliminary volume of interest (VOI) was manually drawn around the primary tumour on each slice (by CC and LM, both experienced in PET/CT segmentation). MTV was then computed automatically using a 42% threshold of the maximum SUV (within the VOI (MTV42%), following established methods [[29], [30], [31]]. In addition to the MTV_42%_, the following frequently reported semi-quantitative PET/CT parameters were extracted from the MTV: SUVmax (maximum SUV in the tumour), SUVpeak (mean SUV of the 3x3x3 voxel region with the highest average SUV), SUVskewness (asymmetry of the SUV histogram), SUVkurtosis (‘peakedness’ of the histogram), SUVentropy (randomness of the histogram), SUVuniformity (homogeneity of the histogram), and total lesion glycolysis (TLG: MTV_42%_*SUVmean).

### Statistical analysis

Statistical analysis was performed with R statistical software (version 2024.09.1). From each pair of continuous variables with a Pearson correlation at least 0.8, the variable with the greatest average correlation with all other variables was excluded from further analyses. Penalised logistic regression models predicting 2-year locoregional failure were obtained using the least absolute shrinkage and selection operator (LASSO) [32]. Seven different models were obtained by using seven pre-defined sets of predictor variables: (1) clinical baseline variables, (2) tumour hr-HPV status, (3) PET/CT variables, (4) clinical baseline variables and tumour hr-HPV status, (5) clinical baseline variables and PET/CT variables, (6) tumour hr-HPV status and PET/CT variables, and (7) clinical baseline variables, tumour hr-HPV status and PET/CT variables combined. Whether patients underwent a radiotherapy boost in addition to the standard treatment, was included in each of the seven models to account for confounding effects. Predictive performance of each model was assessed using the area under the receiver-operating characteristic curve (AUC). In addition to the AUCs based on the full dataset, we calculated bias-corrected AUCs using Harrell’s bootstrap method with 1000 iterations, to account for optimism in model performance estimates and improve generalisability [33].

## Results

### Baseline characteristics

**Fig. 2:**
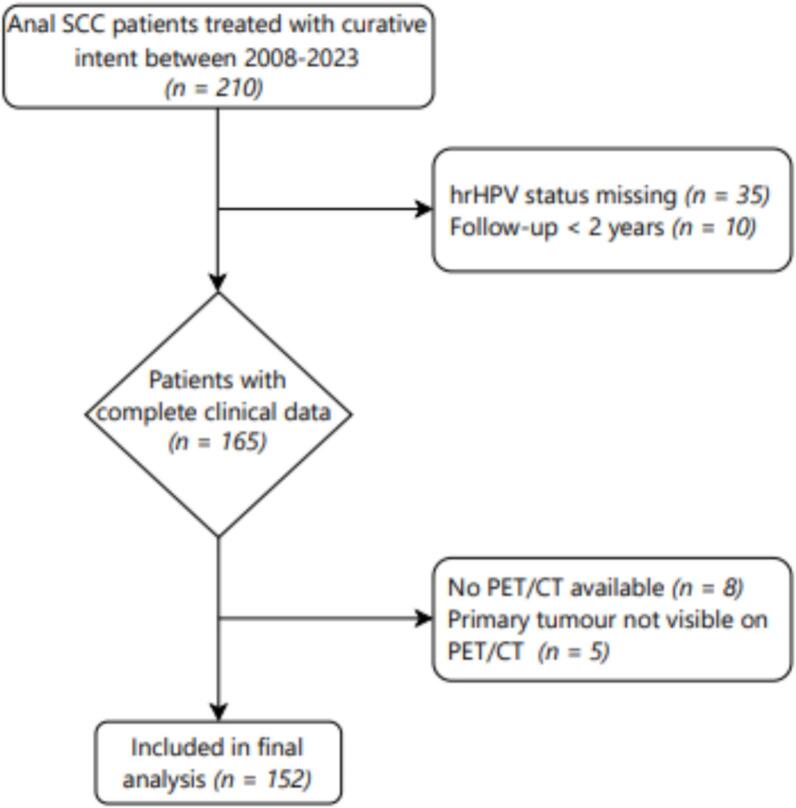
Flowchart of patient selection and exclusions for final analysis.

Between 2008 and 2023, 210 ASCC patients were treated with curative intent cCRT at our institution. A total of 58 patients were excluded for reasons detailed in the in- and exclusion flowchart in Fig. 2, leaving a total study population of 152 patients (34% male, median age 62 years) that met all inclusion criteria. Detailed patient and tumour characteristics for these patients are summarised in Table 1.

**Table 1:** Baseline patient characteristics (n = 152).

**Median age**		62 years (range: 34–88)
**Sex**	Male	52 (34%)
	Female	100 (66%)
**Tumour hr-HPV status**	Negative	15 (10%)
	Positive	137 (90%)
**HIV status**	Negative	82 (54%)
	Positive	9 (6%)
	Unknown	61 (40%)
cT-stage*	cT1	9 (6%)
	cT2	87 (57%)
	cT3	36 (24%)
	cT4	20 (13%)
**Maximum tumour diameter on MRI (median)**		37 mm (range: 0–113 mm)**
cN-stage*	cN0	79 (52%)
	cN+	73 (48%)
**Sequential radiotherapy boost (3x1.8 Gy)**	No	103 (68%)
	Yes	49 (32%)
HPV: human papillomavirus; HIV: human immunodeficiency virus; MRI: magnetic resonance imaging * Staging was performed according to the UICC TNM Classification of Malignant Tumours 8th edition (2017) ** In five patients the primary tumour was not visualised on MRI (resulting in a 0 mm measurement), but detectable on 18F-FDG-PET/CT		

### Oncological outcomes

Overall, 21/152 patients (14%) experienced locoregional treatment failure; 16 patients had residual disease, and 5 patients developed a local recurrence within 2 years following cCRT (within 13–14 months following cCRT completion). Further details regarding the locoregional failures are provided in Table 2. Of the 21 patients with locoregional failure, 18 (86%) had no distant metastases: 17 underwent salvage APR, while 1 patient received palliative treatment due to irresectable disease. The remaining 3 patients presented with both locoregional failure and distant metastases and were managed with palliative care. Of the 17 patients who underwent salvage APR, 4 subsequently developed distant metastases during follow-up. In total, distant metastases were detected in 16 out of 152 patients (11%). Locoregional failure rate was 40% in the hr-HPV-negative group and 11% in the hr-HPV positive group.

**Table 2:** Locoregional failure < 2 years (N = 21).

Locoregional failure < 2 years (N = 21)		
**Locoregional failure**	Residual disease	16 (76%)
	Recurrence	5 (24%)
**Site of failure**	Primary tumour site only	16 (76%)
	Regional nodes only	0 (0%)
	Tumour + regional nodes	5 (24%)
**Distant metastases**		7 (32%)

### Multivariable prediction models

The results of the multivariable regression analysis are summarised in Table 3. Of the initial 8 PET/CT parameters, only MTV42%, SUVskewness, SUVuniformity, SUVkurtosis and TLG were included in the multivariable analysis. The remaining parameters were excluded from further analysis due to strong correlations (ρ ≥ 0.8).

**Table 3:** Multivariable prediction models for locoregional failure within 2 years after cCRT.

**I – Separate models**				
	Selected variables	OR (CI)	AUC-ROC	Bias corrected AUC
**Clinical baseline model**	Age	1.00 (0.94–1.00)	0.62 (0.50–0.73)	0.54 (0.43–0.63)
	Sex (male)	1.00 (1.00–4.75)		
	MRI tumour length	1.00 (1.00–1.03)		
	T-stage (3–4 vs 1–2)	1.00 (0.30–2.31)		
	N-stage (N + )	1.00 (1.00–4.49)		
	Boost (yes)	1.00 (1.00–4.70)		
**hr-HPV model**	hr-HPV positive	0.19 (0.06–1.00)	0.69 (0.57–0.81)	0.67 (0.57–0.76)
	Boost (yes)	2.56 (1.00–6.62)		
**PET/CT model**	MTV42%	1.02 (1.00–1.06)	0.70 (0.58–0.81)	0.64 (0.52–0.74)
	SUVkurtosis	1.00 (0.29–1.04)		
	SUVskewness	1.00 (1.00–32.18)		
	SUVuniformity	1.00 (0–7.8x10^8^)		
	TLG	1.00 (1.00–1.00)		
	Boost (yes)	1.55 (1.00–4.96)		
**II – Combined models**				
	Selected variables	OR (CI)	AUC-ROC	Bias corrected AUC
**Clinical + hr-HPV model**	Age	0.98 (0.94–1.00)	0.73 (0.59–0.86)	0.65 (0.54–0.75)
	Sex (male)	1.21 (1.00–3.79)		
	MRI tumour length	1.00 (0.99–1.03)		
	T-stage (3–4 vs 1–2)	1.00 (0.31–2.65)		
	N-stage (N + )	1.65 (1.00–5.74)		
	hrHPV-positive	0.25 (0.07–1.00)		
	Boost (yes)	1.60 (1.00–5.09)		
**Clinical + PET/CT model**	Age	0.99 (0.94–1.00)	0.70 (0.57–0.83)	0.62 (0.48–0.71)
	Sex (male)	1.47 (1.00–4.52)		
	MRI tumour length	1.00 (0.99–1.03)		
	T-stage (3–4 vs 1–2)	1.00 (0.28–2.22)		
	N-stage (N + )	1.30 (1.00–3.69)		
	MTV42%	1.01 (1.00–1.05)		
	SUVkurtosis	1.00 (0.37–1.11)		
	SUVskewness	1.00 (1.00–21.30)		
	SUVuniformity	1.00 (0–2.8x10^7^)		
	TLG	1.00 (1.00–1.00)		
	Boost (yes)	1.61 (1.00–4.65)		
**hr-HPV + PET/CT model**	hr-HPV positive	0.30 (0.07–1.00)	0.75 (0.65–0.85)	0.69 (0.58–0.77)
	MTV42%	1.01 (1.00–1.05)		
	SUVkurtosis	1.00 (0.39–1.38)		
	SUVskewness	1.00 (1.00–18.73)		
	SUVuniformity	1.00 (1.00–1.8x10^7^)		
	TLG	1.00 (1.00–1.00)		
	Boost (yes)	1.60 (1.00–5.28)		
**All combined**	Age	0.99 (0.94–1.01)	0.73 (0.60–0.86)	0.64 (0.52–0.74)
	Sex (male)	1.21 (0.96–4.12)		
	MRI tumour length	1.00 (0.98–1.02)		
	T-stage (3–4 vs 1–2)	1.00 (0.27–2.50)		
	N-stage (N + )	1.43 (1.00–4.50)		
	hr-HPV positive	0.28 (0.06–1.00)		
	MTV42%	1.01 (1.00–1.04)		
	SUVkurtosis	1.00 (0.53–1.37)		
	SUVskewness	1.00 (1.00–8.84)		
	SUVuniformity	1.00 (0–1.7x10^6^)		
	TLG	1.00 (1.00–1.00)		
	Boost (yes)	1.52 (1.00–4.97)		

Of the “single-modality” predictive models, the hr-HPV status model showed the best overall performance, with an AUC of 0.69 and bias-corrected AUC of 0.67. In this model, hr-HPV positivity was associated with reduced odds of locoregional failure (OR 0.19). The PET/CT only model had a slightly higher AUC (0.70), but after bias correction its performance was more modest (0.64). Within this model, MTV42% (OR 1.02) was the only PET/CT variable with predictive value. The clinical baseline model demonstrated poor predictive ability (bias-corrected AUC 0.54), with no stable predictors identified.

Among the combined models, the best performance was seen in the model combining hr-HPV status and PET/CT parameters (AUC 0.75, bias-corrected AUC 0.69). In this model, hr-HPV positivity (OR 0.30) and MTV42% (OR 1.01) were identified as predictive variables. The clinical and hr-HPV model showed a slightly lower performance (bias-corrected AUC 0.65), with N-stage (OR 1.65) and hr-HPV positivity (OR 0.25) emerging as relevant predictors. The model combining clinical, hr-HPV and PET/CT variables showed a bias-corrected AUC of 0.64.

Whether patients received a sequential boost of radiotherapy was identified as a confounding factor (predictor for treatment failure) in all models with ORs ranging between 1.00–2.56.

## Discussion

In this study, we explored the value of combining clinical characteristics and image-based staging variables with hr-HPV status and semi-quantitative PET/CT parameters, to develop models for predicting locoregional failure within two years after cCRT in patients with ASCC. Overall, tumour hr-HPV status was found to be the strongest predictor, with a markedly higher rate of locoregional failure among patients with hr-HPV negative tumours compared to the hr-HPV-negative subgroup (40% versus 11%). The model combining hr-HPV status and MTV42% derived from PET/CT demonstrated the best predictive performance, although the difference in performance compared to the model including only hr-HPV status was only small (bias corrected AUC 0.69 versus 0.67).

A previous study by Rusten et al. currently represents the only published model integrating both pre-treatment 18F-FDG-PET/CT parameters and clinicopathological risk factors for predicting locoregional failure in anal cancer [18]. In their cohort of 93 patients treated with cCRT, MTV, TLG, hr-HPV negativity, and advanced nodal status were each associated with locoregional failure in both univariable and bivariable analyses. Notably, the combination of hr-HPV negativity and advanced nodal stage demonstrated the strongest predictive value, outperforming PET/CT parameters alone. Our findings confirm hr-HPV status as an important prognostic variable. Unlike Rusten et al., who focused on univariable and bivariable associations, we systematically compared multiple multivariable models incorporating different sets of predictors. This approach allowed us to quantify the added value of PET/CT-derived parameters more directly. To address the risk of overfitting – which is particularly relevant in settings with few events – we applied bootstrap-based bias correction, thereby providing a more robust estimate of model performance. Two prospective studies focused solely on pre-treatment PET/CT-derived metrics and – in line with our findings – also identified pre-treatment MTV as the only PET/CT parameter associated with recurrence [[24], [34]]. Given the low incidence of anal cancer, existing studies, including ours, remain underpowered for external validation. To advance predictive modelling in this field, multi-institutional collaboration will be essential to enable pooled analyses and prospective validation within clinical trials.

From a clinical perspective, pre-treatment prediction of locoregional failure in anal cancer can be helpful to explore more personalised treatment strategies. Currently, national guidelines in the Netherlands advocate a relatively high dose of radiotherapy (at least 50.4 Gy to macroscopic disease and 30–36 Gy to elective regions) for all patients, regardless of individual risk (3). At our institution an additional boost of three fractions of 1.8 Gy to the GTV is already routinely prescribed to patients with clinically detectable gross residual tumour in the fifth week of radiation therapy (after 45 Gy), determined via interim clinical evaluation and MRI. In our current cohort, approximately one third of patients received this boost. These patients constitute the subgroup in our cohort that showed the poorest clinical response already after five weeks of treatment suggesting a more aggressive or relatively radiation-resistant tumour biology. This may also explain their higher risk of locoregional failure (with ORs up to 2.56). The recently published PLATO-ACT4 trial has demonstrated that in patients with early-stage anal cancer, a reduced dose of chemoradiotherapy can achieve comparable disease control with fewer side effects [14]. These findings support the feasibility of dose de-escalation in selected low-risk patients, thereby reducing treatment-related morbidity. At the same time, identifying patients at higher risk of locoregional failure using pre-treatment parameters from baseline staging could allow for selective dose escalation or intensified surveillance. This risk-adapted approach would represent a significant shift from the current “one-size-fits-all” paradigm towards a more individualised, biology-driven model of care. Furthermore, the role of interim response evaluation in the fifth week of radiation therapy (after 45 Gy), although routinely used in some centers like ours, remains poorly documented in the literature and warrants further investigation. Beyond baseline risk stratification, interim evaluations may prove valuable in guiding adaptive strategies such as treatment intensification or early modification. Some preliminary reports have already shown promise for sequential MRI evaluations to predict and monitor response in anal cancer [[35], [36], [37]].

Another interesting topic for future studies would be to investigate the potential complementary role of quantitative MRI evaluation. In other pelvic malignancies, including rectal cancer, several studies have shown that quantitative metrics derived from MRI including functional sequences (in particular diffusion-weighted imaging) can have value to predict local treatment outcomes [[38], [39]]. In our study, it was not deemed feasible to include quantitative MRI parameters as many patients were referred from other institutions and MRIs (when available) were performed according to variable local protocols. This variation in image acquisition is known to significantly impact quantitative image models [40]. However, we did include several clinical MRI-derived parameters in our models, including tumour length and cTN-stage. Future studies with standardised acquisition protocols are needed to further explore the potential added value of MRI in predictive modeling and treatment personalisation.

Our study has some limitations, in addition to its retrospective nature and the issues addressed in the previous sections. Although our single-center patient cohort is one of the largest in its kind, the total number of patients (and locoregional failures) remained relatively small owing to the low incidence of anal cancer. This limited event rate restricted the number of predictors that could be included in our models to avoid overfitting. Additionally, we had to rely on statistical methods to estimate model generalisability as it was not feasible to split the dataset into separate training and validation sets. Furthermore, given the long inclusion period (2008–2023), patients were staged according to different AJCC/UICC TNM editions. As nodal subcategories are defined differently across staging systems, bombined with small subgroup sizes, further nodal stratification was not feasible and nodal status was therefore dichotomised (N0 vs N + ). In addition, previous studies have reported that HIV positivity is associated with poorer treatment outcomes [41]. However, HIV status was only available for a limited number of patients within our cohort, precluding meaningful analyses. Similarly, although serum SCCA is a potentially relevant biomarker, baseline pre-treatment values were not consistently available across the cohort and were therefore not included in the analyses. Future prospective studies should aim to systematically collect baseline serum SCCA alongside other potential emerging blood-based biomarkers, such as circulating tumour DNA (ctDNA) and plasma HPV, to further explore their complementary predictive value in risk stratification models.

In conclusion, this study shows that a multivariable model combining hr-HPV status and semi-quantitative PET/CT parameters offers moderate predictive performance for locoregional failure after cCRT in patients with ASCC. Among the variables assessed, hr-HPV negativity emerged as the strongest independent predictor. These findings highlight the potential value of integrating biological and imaging markers for baseline risk stratification aiming to guide more personalised treatment strategies. Future prospective studies with external validation, pooling data from multiple institutions, are needed to confirm these findings and to evaluate their clinical applicability.

## CRediT authorship contribution statement

**C. Ceuppens:** Writing – review & editing, Writing – original draft, Visualization, Formal analysis, Data curation, Conceptualization. **L.A. Min:** Writing – review & editing, Writing – original draft, Visualization, Data curation, Conceptualization. **B. van Triest:** Writing – review & editing, Visualization, Supervision, Conceptualization. **M. Lopez-Yurda:** Writing – review & editing, Methodology, Formal analysis. **W.V. Vogel:** Writing – review & editing, Visualization, Supervision, Conceptualization. **P. Snaebjornsson:** Writing – review & editing, Visualization, Conceptualization. **M. Maas:** Writing – review & editing, Visualization, Conceptualization. **R.G.H. Beets-Tan:** Writing – review & editing, Visualization, Conceptualization. **G.L. Beets:** Writing – review & editing, Visualization, Conceptualization. **B.A. Grotenhuis:** Writing – review & editing, Visualization, Supervision, Conceptualization. **D.M.J. Lambregts:** Writing – review & editing, Visualization, Supervision, Conceptualization.

## Declaration of competing interest

The authors declare that they have no known competing financial interests or personal relationships that could have appeared to influence the work reported in this paper.
